# A Rare and Aggressive Case of Malignant Insulinoma

**DOI:** 10.7759/cureus.73719

**Published:** 2024-11-15

**Authors:** Francisco Gonçalves, Daniela Duarte, Catarina R Silva

**Affiliations:** 1 Department of Internal Medicine, Centro Hospitalar Tondela-Viseu, Viseu, PRT

**Keywords:** malignant insulinoma, multidisciplinary management, pancreatic neuroendocrine tumors, transarterial chemoembolization (tace), tumor-induced hypoglycemia, whipple's triad

## Abstract

Insulinomas are rare pancreatic neuroendocrine tumors (NETs) characterized by autonomous insulin secretion leading to hypoglycemia. Malignant insulinomas are defined by the presence of metastases and present significant therapeutic challenges due to limited treatment options.

We report the case of a 69-year-old woman with a two-month history of neuroglycopenic symptoms, including morning headaches, blurred vision, palpitations, and sweating, which were alleviated by sugar intake. An episode of severe hypoglycemia-induced unconsciousness necessitated intramuscular glucagon administration, resulting in regained consciousness. The combination of neuroglycopenic symptoms relieved by carbohydrate and documented hypoglycemia fulfilled Whipple's triad, prompting evaluation for an insulin-secreting tumor.

Laboratory findings revealed elevated endogenous insulin and C-peptide levels, indicating hyperinsulinemia. Imaging studies, including contrast-enhanced computed tomography (CT) and magnetic resonance imaging (MRI), identified a 25 mm solid lesion in the pancreatic body and multiple hepatic metastases. A ⁶⁸Ga-DOTA-NOC positron emission tomography (PET) scan demonstrated high somatostatin receptor expression in both the pancreatic lesion and hepatic metastases. Ultrasound-guided liver biopsy revealed a high-grade (G3) neuroendocrine carcinoma with a Ki-67 proliferation index exceeding 30%, confirming the diagnosis of malignant insulinoma.

Multidisciplinary consultation recommended initiation of systemic chemotherapy with cisplatin and etoposide. Despite optimized medical management, including dextrose infusion, diazoxide, octreotide, and corticosteroids, the patient experienced persistent severe hypoglycemia. Transarterial chemoembolization (TACE) of the tumor vasculature was performed to mitigate hypoglycemia by reducing tumor burden. Post-procedure, the patient developed a cerebellar hemorrhage leading to coma and subsequent death.

This case underscores the aggressive nature and poor prognosis associated with malignant insulinomas, particularly those with high proliferative indices. It highlights the complexities of managing refractory hypoglycemia in the context of widespread metastatic disease and emphasizes the urgent need for effective therapeutic strategies to improve patient outcomes.

## Introduction

Insulinomas are rare pancreatic neuroendocrine tumors (NETs) originating from β-cells of the islets of Langerhans, characterized by autonomous and excessive insulin secretion, leading to hypoglycemia [1]. The estimated incidence of insulinomas is approximately 1-4 cases per million person-years [2,3].

Insulinomas predominantly originate within pancreatic parenchyma, with approximately 90% of these neuroendocrine tumors localized to the head and body regions of the pancreas [4].

The distinction between benign and malignant insulinomas is often ambiguous, due to the lack of definitive histopathological criteria and the reliance on the presence of metastases for classification [5]. Therefore, non-metastatic insulinomas are frequently referred to as indolent, whereas those exhibiting metastatic behavior are termed aggressive [5-7]. A comprehensive 60-year retrospective study at the Mayo Clinic reported that malignant insulinomas constitute approximately 6% of all insulinoma cases [3].

The incidence of insulinomas exhibits an age-specific peak occurring in the fifth decade of life among male patients and in the sixth decade among female patients. Epidemiological studies have demonstrated that the overall incidence is slightly higher in women compared to men [8].

Clinically, insulinomas manifest through hypoglycemic episodes resulting from unregulated insulin secretion. The diagnostic cornerstone is Whipple's triad, which encompasses (1) symptoms consistent with hypoglycemia, (2) documented low plasma glucose concentration during symptoms, and (3) amelioration of symptoms upon glucose normalization [9,10]. Hypoglycemic symptoms are categorized into neuroglycopenic symptoms, such as confusion, visual disturbances, seizures, or loss of consciousness, and autonomic symptoms, including diaphoresis, palpitations, tremors, and hunger [9].

While benign insulinomas have been extensively studied concerning their epidemiology, clinical presentation, and management strategies, malignant insulinomas remain poorly understood due to their rarity [11]. The optimal management of malignant insulinomas is challenging, with no standardized treatment protocols established [11]. Therapeutic options are often limited and primarily focus on controlling hypoglycemia and reducing tumor burden.

This case report aims to elucidate the diagnostic process, therapeutic interventions, and clinical outcomes in a patient with malignant insulinoma presenting with severe refractory hypoglycemia. It underscores the critical importance of a multidisciplinary approach in managing this rare and aggressive neoplasm, highlighting the complexities and challenges faced in clinical practice.

This case report was previously presented as a poster at the 29th Portuguese Internal Medicine National Congress, held in Porto, Portugal, from May 4 to 7, 2023.

## Case presentation

A 69-year-old female patient presented to her primary care physician with a two-month history of headaches, blurred vision, palpitations, and sweating, predominantly in the morning, which resolved after ingestion of sugar or honey. Her medical history was significant for depressive syndrome and dyslipidemia, for which she was on a tricyclic antidepressant, benzodiazepine, and statin.

During an episode of palpitations and sweating at her physician's office, hypoglycemia was documented, prompting the initiation of glycemic monitoring using a continuous glucose monitoring system. Because of this, she was admitted to the emergency department after being found unconscious with severe hypoglycemia (21 mg/dL). She regained consciousness after intramuscular administration of glucagon.

She reported persistent hypoglycemic episodes the night before, despite substantial sugar intake. Laboratory investigations revealed mild hyponatremia (130 mEq/L) and a capillary blood glucose of 63 mg/dL. A dextrose infusion was initiated. Given the clinical presentation consistent with Whipple's triad, an abdominal-pelvic computed tomography (CT) scan with contrast was performed.

**Figure 1:**
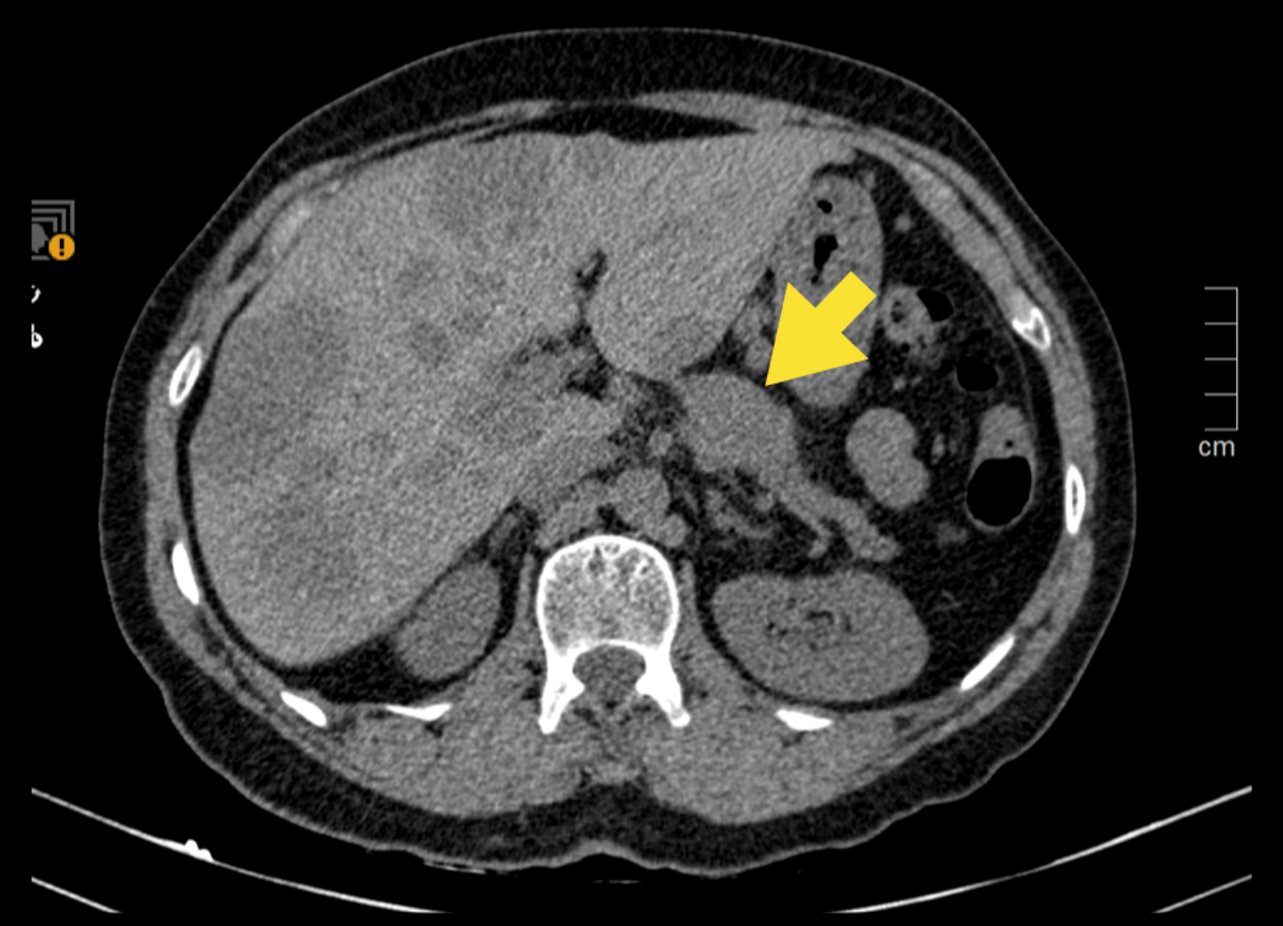
Axial abdominal-pelvic CT scan Liver with lobulated contours and multiple hypodense nodular lesions and a hypodense area in the body region of the pancreas (yellow arrow) CT: computed tomography

CT imaging revealed a liver with lobulated contours and multiple hypodense nodular lesions featuring central areas of greater hypodensity and peripheral enhancement in both the left and right lobes. The largest lesion measured 57 mm and was indicative of secondary metastatic lesions. The pancreas exhibited regular contours but showed a slightly hypodense area in the body region measuring 21 mm, suggesting a probable solid lesion (Figure 1).

**Figure 2:**
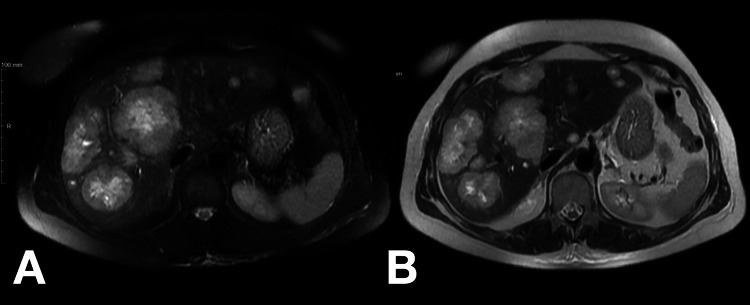
Abdominal MRI showing multiple nodules compatible with liver metastasis T2 fat-suppressed (A) and T2-weighted MRI (B) images showing multiple heterogeneous nodules on the liver parenchyma consistent with metastatic lesions MRI: magnetic resonance imaging

**Figure 3:**
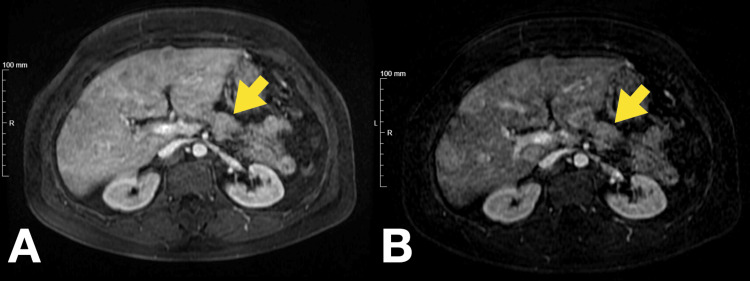
Abdominal MRI showing multiple hypervascular nodules compatible with liver metastasis and hypervascular nodule in the pancreatic body Arterial phase T1 fat-suppressed images showing multiple hypervascular enhancing nodules on the liver consistent with metastatic lesions and a solid enhancing nodule in the pancreatic body, as the primary lesion, compatible with NET MRI: magnetic resonance imaging, NET: neuroendocrine tumor

**Figure 4:**
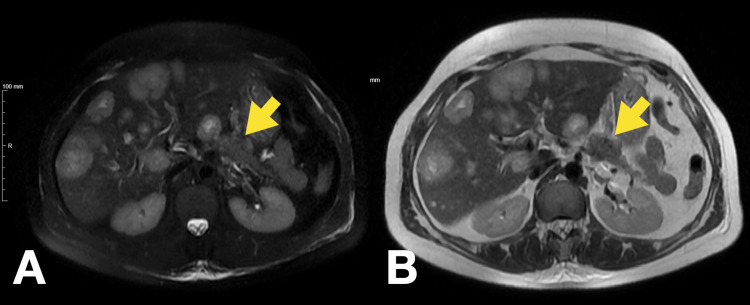
Abdominal MRI showing multiple liver nodules and solid nodular lesion of the pancreatic body T2 fat-suppressed (A) and T2-weighted MRI (B) images showing NET (yellow arrow) and liver metastases MRI: magnetic resonance imaging, NET: neuroendocrine tumor

For further evaluation and management of severe symptomatic hypoglycemia, the patient was admitted. An abdominal magnetic resonance imaging (MRI) scan described a globular liver, slightly enlarged, with a markedly heterogeneous structure due to multiple hypervascular nodules consistent with metastatic deposits (Figure 2). The largest nodule was located on the superior convexity of segment VIII and measured 65 mm in its greatest dimension. There was atrophy of the pancreatic tail and part of the body, with a solid lesion identified in the pancreatic body measuring 25 mm, compatible with a suspected NET (Figure 3 and Figure 4).

Laboratory results showed elevated insulin and C-peptide levels, while parathyroid hormone levels were within the normal range, corroborating the clinical suspicion (Table 1).

**Table 1:** Summary of laboratory results

Test	Result	Normal range
Insulin	117.60 μU/mL	2.60-37.60 μU/mL
Parathyroid hormone	76.40 pg/mL	18.50-88.00 pg/mL
C-peptide	5.49 ng/mL	0.81-3.85 ng/mL

A malignant insulinoma was suspected based on clinical, laboratory, and imaging findings. An ultrasound-guided liver biopsy was performed without complications. Liver biopsy histopathology revealed a high-grade (G3) neuroendocrine carcinoma with a Ki-67 proliferation index exceeding 30%, thereby confirming the diagnosis of malignant insulinoma.

**Figure 5:**
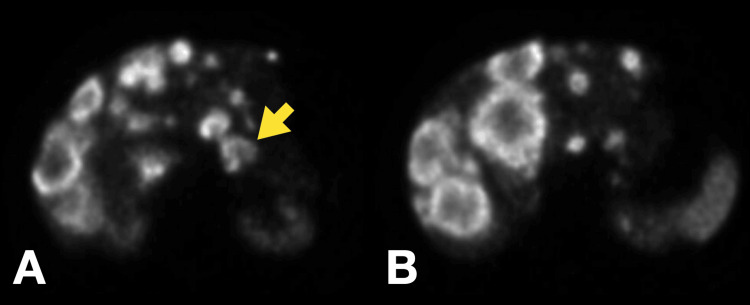
PET scan using 68Ga-DOTA-NOC PET scan using ^68^Ga-DOTA-NOC shows a nodular formation in the pancreatic body consistent with a neuroendocrine tumor (yellow arrow) and multiple lesions on the liver compatible with widespread metastasis PET: positron emission tomography

A positron emission tomography (PET) scan using ^68^Ga-DOTA-NOC showed a nodular formation with irregular contours and central calcification in the pancreatic body, measuring approximately 30×28 mm. The lesion exhibited a high uptake of ^68^Ga-DOTA-NOC, consistent with a neuroendocrine tumor with high somatostatin receptor expression in the pancreas. An area of irregular density between the left hepatic lobe and the stomach also showed increased radiotracer uptake, suggesting metastatic disease with elevated receptor expression. The liver demonstrated multiple lesions with marked increased uptake of ^68^Ga-DOTA-NOC, extensively occupying the parenchyma and indicating widespread metastasis with high somatostatin receptor expression. The largest lesion was centered around segments IV/VIII, measuring approximately 68×65 mm, showing significant peripheral uptake and a hypoactive center (Figure 5).

A multidisciplinary team meeting including internal medicine, general surgery, medical oncology, and interventional radiology specialists concluded that given the extensive hepatic metastases, systemic chemotherapy with cisplatin and etoposide should be initiated.

During chemotherapy, the patient continued to experience severe hypoglycemia despite optimized medical therapy, which included continuous dextrose infusion, diazoxide, octreotide, and corticosteroids. Due to persistent hypoglycemia and the impracticality of surgical resection of the insulinoma, interventional radiology performed transarterial chemoembolization (TACE) of the tumor vasculature. The intervention was completed without immediate complications.

**Figure 6:**
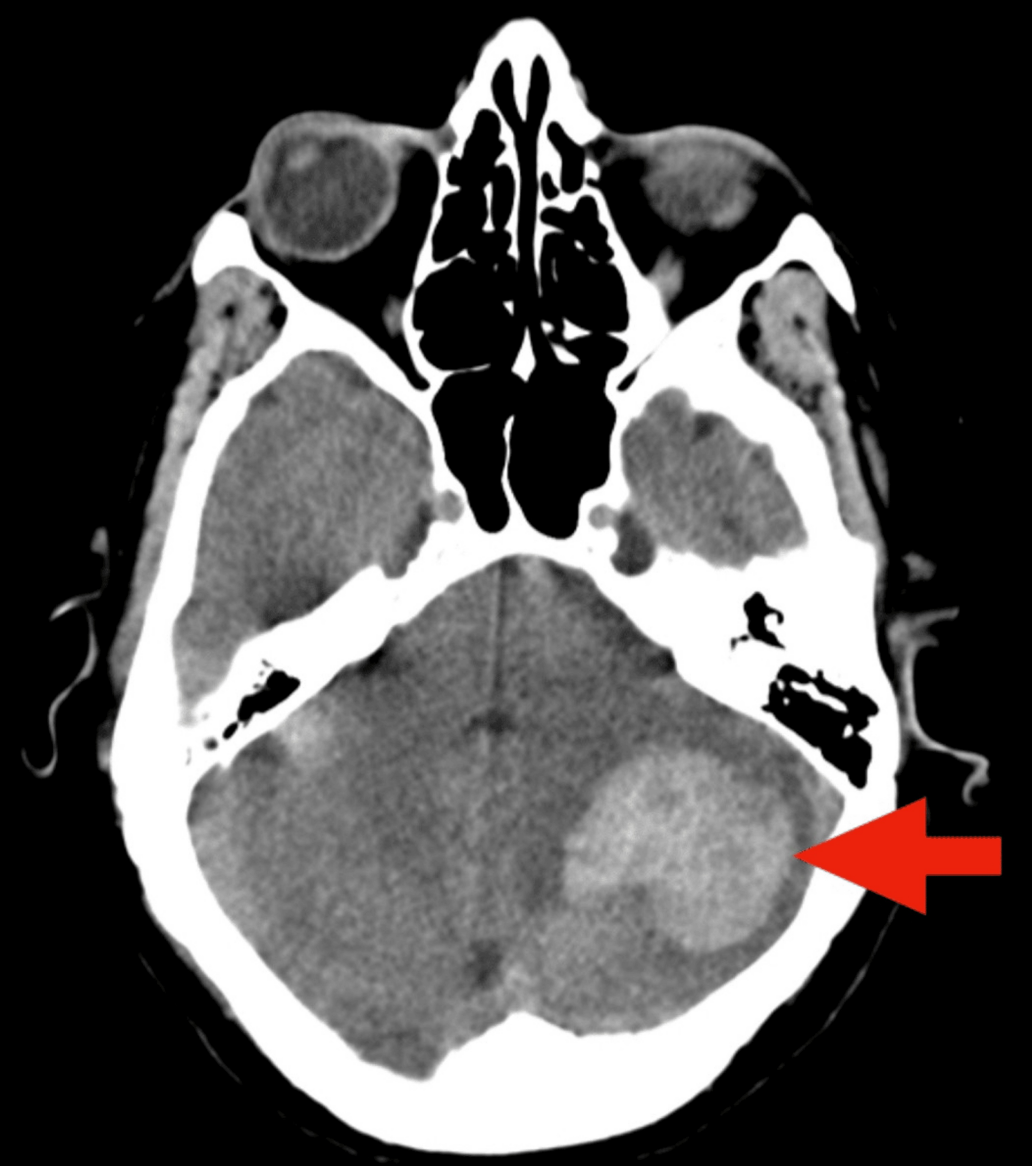
Non-contrast cranial CT scan CT scan showing spontaneous hyperdensity in the left cerebellar cortico-subcortical area compatible with hemorrhagic lesion (red arrow) CT: computed tomography

The following day, the patient became comatose, exhibiting signs of a hypertensive crisis, bradycardia, and respiratory insufficiency. She was promptly intubated and provided with ventilatory support. An urgent non-contrast CT scan revealed a left cerebellar cortico-subcortical hemorrhagic lesion measuring 34×37 mm (Figure 6).

An emergency decompressive craniotomy was performed by neurosurgery. She was admitted to the intensive care unit with a labetalol infusion but unfortunately passed away later that day.

## Discussion

This case highlights the diagnostic challenges and complexity of managing a patient with malignant insulinoma, an exceedingly rare pancreatic NET.

The patient's presentation with symptoms of hypoglycemia such as headaches, blurred vision, palpitations, and sweating, particularly in the morning and alleviated by sugar intake, is classic for hyperinsulinemic hypoglycemia. The confirmation of Whipple's triad (neuroglycopenic symptoms, confirmed low plasma glucose levels during symptoms, and prompt relief of symptoms following the consumption of glucose) solidified the clinical suspicion of an insulin-secreting tumor [9,10].

The neuroglycopenic symptoms, including unconsciousness despite high sugar intake, underscore the severity of the hypoglycemia and the potential for rapid deterioration. In this case, the prompt intramuscular administration of glucagon in the prehospital setting was lifesaving.

Epidemiological investigations consistently reveal that the median age at diagnosis for insulinoma is in the fifth decade of life among male patients and in the sixth decade among female patients, with a modestly higher overall incidence observed in females compared to males [8]. This demographic distribution is exemplified by the current case of a 69-year-old female, whose advanced age at presentation is in concordance with the established prevalence data. Such findings underscore the importance of considering age and gender-specific trends in the clinical evaluation and diagnostic approach to insulinoma.

Biochemical analysis revealed markedly elevated insulin and C-peptide levels, indicative of endogenous hyperinsulinemia. Imaging studies, including contrast-enhanced CT, MRI, and ^68^Ga-DOTA-NOC PET scans, localized a pancreatic body lesion with extensive hepatic metastases exhibiting high somatostatin receptor expression. The imaging characteristics are consistent with a well-differentiated NET, yet the high Ki-67 index (>30%) from the liver biopsy indicates a high-grade neuroendocrine carcinoma, reflecting aggressive behavior and poor prognosis.

Medical management of hypoglycemia includes frequent glucose administration, diazoxide to inhibit insulin release, octreotide to suppress hormone secretion, and corticosteroids to antagonize insulin action [9]. In this case, despite optimized medical therapy, the patient continued to experience severe hypoglycemia.

Surgical resection is the mainstay for localized insulinomas; however, in the context of extensive hepatic metastases, curative surgery is often not feasible [11].

In this case, to complement the medical management and the inability to perform surgical resection, systemic chemotherapy was initiated with cisplatin and etoposide, based on the high-grade nature of the tumor and its extensive metastasis. Platinum-based regimens have shown some efficacy in high-grade neuroendocrine carcinomas, although response rates are variable and often short-lived [5]. The persistence of hypoglycemia despite chemotherapy led to the consideration of locoregional therapies. TACE was employed to reduce tumor burden by delivering chemotherapeutic agents directly to the tumor vasculature while inducing ischemia, potentially reducing insulin secretion [12]. TACE can provide symptomatic relief and control hormone secretion in functional NETs, although evidence is limited and primarily derived from hepatic metastasis management [12].

The acute deterioration following TACE, culminating in cerebellar hemorrhage and death, is a tragic outcome. This underscores the importance of close monitoring and risk assessment when employing invasive interventions in patients with advanced NETs.

This case highlights the complex management of malignant insulinomas, which requires a multidisciplinary approach. The aggressive nature of high-grade NET necessitates prompt and tailored interventions, balancing potential benefits against risks.

## Conclusions

This case elucidates the intricate challenges inherent to the diagnosis and management of malignant insulinomas, a rare and aggressive variant of pancreatic NETs. The patient's clinical presentation, characterized by severe and refractory hypoglycemia, underscores the critical importance of recognizing Whipple's triad in the early identification of insulin-secreting neoplasms.

Despite the deployment of a comprehensive multidisciplinary approach, the rapid progression to extensive hepatic metastases and the subsequent fatal cerebral hemorrhage highlight the aggressive biology and poor prognosis associated with malignant insulinomas. The failure of conventional medical therapies, including systemic chemotherapy with cisplatin and etoposide, and locoregional interventions such as TACE, underscores the urgent need for more effective therapeutic strategies.
